# How can sarcopenia be prevented after gastrointestinal surgery?

**DOI:** 10.1002/ags3.12617

**Published:** 2022-09-05

**Authors:** Yukinori Kurokawa

**Affiliations:** ^1^ Department of Gastroenterological Surgery Osaka University Graduate School of Medicine Osaka Japan

Sarcopenia is a morbid condition characterized by loss of skeletal muscle mass and strength. It has long been known that sarcopenia leads to physical disability and poor quality of life, and it also negatively affects postoperative outcomes in patients with malignancies. In the field of resectable gastric cancer, for instance, Taniguchi et al reported that a preoperative low psoas muscle index (PMI) measured by cross‐sectional computed tomography (CT) at the umbilical level was a significant risk factor for postoperative pneumonia and an independent prognostic factor for recurrence‐free survival in patients who underwent gastrectomy.^1^ Hashimoto et al reported that patients with a low PMI had a higher incidence of hematological and nonhematological toxicities during preoperative chemotherapy for gastric cancer than those with a high PMI.^2^ Sarcopenia may reduce the metabolic rate or the clearance of preoperative chemotherapeutic drugs, and may therefore result in higher concentrations of these drugs in the blood.

In this issue of the *Annals of Gastroenterological Surgery*, Sugimura et al reported that the preoperative skeletal muscle index (SMI) measured with multifrequency bioelectrical impedance was associated with overall survival, but not with the incidence of postoperative complications after esophageal cancer surgery.^3^ Interestingly, measurement of the preoperative 6‐min walk distance (6MWD) as an indicator of physical performance significantly predicted both postoperative pneumonia and survival. The combination of the SMI and 6MWD could stratify overall survival more clearly than the use of either one alone. Daitoku et al analyzed the association between the preoperative SMI measured with cross‐sectional CT images at the L3 level and the densities of tumor‐infiltrating lymphocytes (TILs) assessed by immunohistochemistry in patients who underwent colorectal cancer surgery, and reported that the preoperative SMI was significantly correlated with the density of CD3^+^ and CD8^+^ cells.^4^ Although it remains unclear if sarcopenia is a cause or result of the low density of TILs, the close relationship may contribute to poor posttreatment outcomes in patients with both malignancies and sarcopenia.

As noted above, many studies have indicated that sarcopenia leads to poor treatment outcomes in patients with gastrointestinal malignancies. One remaining issue is that there is no standard, universal method to screen for sarcopenia in patients with malignancies, because previous studies used different methods to diagnose sarcopenia. Prospective, large‐scale, multi‐institutional studies are needed to determine the most useful screening method for sarcopenia. Another important issue is how to prevent the postoperative onset or aggravation of sarcopenia in patients with malignancies. Unfortunately, most previous studies did not outline any solutions. Preoperative strength training usually requires a delay of surgery, while postoperative strength training may not be feasible among patients who have undergone cancer surgery. In Japan, ~60% of patients who undergo gastric or colorectal surgery are elderly (≥70 y).^5^ Can any drugs or medical equipment effectively increase skeletal muscle mass and strength? I strongly expect that studies presenting solutions to this issue will be reported in the *Annals of Gastroenterological Surgery* in the near future.

## DISCLOSURE

Conflict of Interest: The author declares no conflicts of interest for this article.
